# Dielectrophoretic deposition of palladium-modified zinc oxide nanowires for high-performance humidity sensors

**DOI:** 10.1039/d6ra05014j

**Published:** 2026-10-01

**Authors:** Kainat Farooq, Muhammad Tayyab Zahoor, Zahid Imran, Waqqar Ahmed

**Affiliations:** a Materials Laboratory, Department of Physics, COMSATS University Islamabad Park Road 45500 Islamabad Pakistan w.ahmed@comsats.edu.pk; b Catalysis and Sensing Materials Laboratory, Department of Physics, COMSATS University Islamabad Park Road 45500 Islamabad Pakistan

## Abstract

Semiconductor–metal heterostructures possess great potential for humidity sensing applications due to the synergistic electronic effects arising from the combination of the two materials. However, the fabrication of such heterostructures and the development of efficient devices demand the precise deposition of nanostructures between planar electrodes, which remains a considerable challenge. In this study, we address these challenges by first synthesizing Pd modified ZnO nanowires (NWs) and subsequently employing dielectrophoresis (DEP) to achieve their controlled deposition between planar electrodes, thereby enabling the realization of an efficient sensing device. ZnO NWs were synthesized *via* a hydrothermal method and subsequently modified with Pd nanoparticles (NPs) through sonochemical synthesis. The resulting Pd-coated ZnO NWs were effectively deposited between conducting electrodes using DEP, ensuring well-bridged electrode gaps. The higher concentration of NWs, combined with the synergistic Pd–ZnO heterostructure effects, resulted in remarkable sensing characteristics. The device exhibited a reduced hysteresis of 5.5%, superior response and recovery times of 5 s and 8 s, respectively, and a significantly enhanced sensitivity of 54% toward humidity. These findings highlight the effectiveness of dielectrophoretic deposition for assembling semiconductor–metal nanostructures and demonstrate its potential in the fabrication of highly sensitive humidity sensors, opening promising avenues for future advancements in environmental monitoring and related applications.

## Abstract 2

Pd-modified ZnO nanowires were assembled between planar electrodes using dielectrophoresis to fabricate an efficient humidity-sensing device. The synergistic Pd–ZnO heterostructure demonstrated excellent humidity sensitivity and sensing performance.

## Graphical abstract

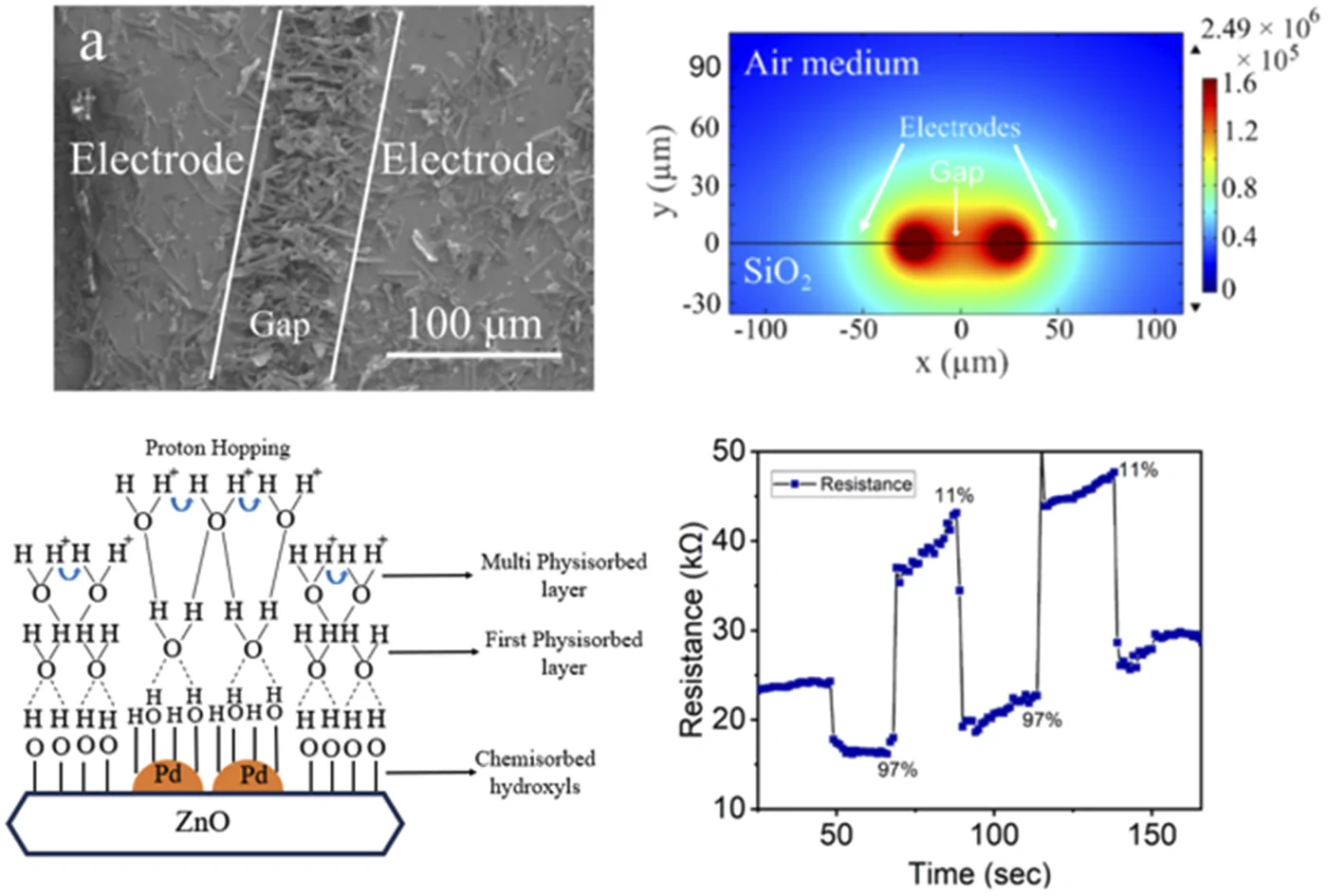

## 1. Introduction

Humidity sensors have gained great attention recently due to their wide range of environmental and industrial applications. Humidity has a profound influence across diverse industries including agriculture, pharmaceuticals, chemicals, textiles, food processing, healthcare, scientific exploration, and environmental control.^1–3^ Generally, metal oxide semiconductors like ZnO, SnO_2_, TiO_2_, CuO, WO_3_, and Al_2_O_3_ are employed as humidity sensors.^4^ Among the metal oxides, ZnO is especially attractive owing to its extraordinary properties, such as substantial variations in resistance with water absorption, ease of fabrication, stable performance across varied temperature and humidity levels, chemical inertness, and cost-effective synthesis.^5,6^ One-dimensional ZnO nanostructures not only have a high surface-to-volume ratio but also contain abundant surface vacancies, providing sufficient active sites for chemisorption. This characteristic makes them highly sensitive and selective to changes in ambient conditions.^6–8^

Despite their widespread use, the traditional humidity sensors based only on pure ZnO face limitations due to their poor linearity, and restrained sensitivity to water molecules resulting from their hydrophobic nature.^9,10^ Humidity sensing principally occurs at the surface, enhancing sensor qualities such as sensitivity, response time, recovery time, accuracy, and chemical stability can be achieved through surface morphology engineering.^9,10^ One way in which the sensing capability of ZnO based sensor can be improved is to make a heterostructure of ZnO with noble metals.^11^ This enhancement in sensor performance is typically explained through either a chemical mechanism,^12^ or an electronic mechanism.^13^

Among noble metals, Pd is a good choice due to its interesting electronic and catalytic properties. With the incorporation of Pd on ZnO NWs, a Schottky junction is expected to form, facilitated by their disparate work functions as Pd possess relatively high work function compared to the ZnO.^14^ Consequently, electrons are expected to transfer from the ZnO NWs to the Pd, resulting in upward band bending and the formation of an electron-depletion region in ZnO near the Pd/ZnO interface. This interfacial charge redistribution increases the potential barrier and contributes to an increase in the baseline resistance of the Pd–ZnO heterostructure.^15^ Upon exposure to increasing humidity, however, water molecules adsorb onto the ZnO surface and interact with surface-active sites, leading to the formation of hydroxyl and protonic species. These processes can modulate the surface charge and reduce the effective electron-depletion region, while the resulting protonic species provide an additional pathway for charge transport.^16^ In particular, Pd can facilitate the dissociation of adsorbed water molecules owing to its catalytic activity, thereby increasing the availability of protonic charge carriers and promoting proton transport through a Grotthuss-type mechanism.^16,17^

While Pd incorporation improves the electronic properties of ZnO NWs for humidity detection and water dissociation, realizing their full sensing potential requires careful optimization of NW deposition. A resistivity-based humidity sensor typically relies on a humidity-sensitive material, such as NWs, deposited on planar electrodes. Consequently, optimizing the deposition of NWs can markedly enhance the sensor's humidity-sensing capabilities. A higher concentration of NWs leads to a higher surface area available for the adsorption of water molecules. This increase in adsorption sites is crucial as it significantly boosts the sensor's sensitivity, enabling more accurate and efficient humidity measurements.

Herein, we show that DEP is a highly effective approach for fabricating humidity sensing devices incorporating Pd–ZnO NWs. The large number of NWs within the electrode gap, combined with the synergetic effects provided by the presence of Pd on ZnO, the device demonstrated superior humidity sensing capabilities.

## 2. Experimental

### 2.1 Synthesis

ZnO NWs were synthesized using the hydrothermal method. During synthesis of ZnO NWs, zinc nitrate hexahydrate (Zn(NO_3_)_2_·6H_2_O) was used as a precursor, and urea (CO(NH_2_)_2_) was used as a stabilizer. This procedure consists of dissolving 0.04466 g of Zn(NO_3_)_2_·6H_2_O in 15 mL of deionized water and 0.12012 g of CO(NH_2_)_2_ in 200 mL of deionized water to make 0.01 M solutions. Moreover, an autoclave bottle was used to combine both molar solutions. After that, the solution was placed in the furnace for about 6 hours at the temperature of 120 °C and then was cooled at room temperature. ZnO NWs were allowed to settle down. The supernatant was removed from the bottle once all the particles had settled down to the bottom. The product was repeatedly cleaned using the decantation method to get rid of impurities. Coating of these ZnO NWs with Pd NPs was the next step after their synthesis.

The sonochemical method was employed for synthesis of Pd NPs modified ZnO NWs. Primarily, 12 mL of 0.1 mM H_2_PdCl_4_ solution was prepared in ethanol. Subsequently, this mixture was combined with an already prepared 3 mL ZnO NWs solution. After mixing, it was given around 45 min in a sonicator. Within 45 min, the white-colored ZnO NWs turned brown, indicating the modification of Pd NPs on ZnO NWs. To allow the modified NWs to settle, the solution was then left undisturbed for 1 hour. The product was then repeatedly cleaned to get rid of contaminants after the colorless supernatant was removed, and finally the Pd–ZnO NWs were dispersed in DI water for the deposition experiments.

### 2.2 Device fabrication

Shadow masking technique was employed to fabricate coplanar electrodes on a glass substrate, as reported in our earlier work.^18^ A humidity sensing device was fabricated using electric field assisted deposition of Pd–ZnO NWs. For the DEP experiment, a 15 V voltage and 1 MHz frequency were used. A function generator was used to apply the AC voltage and frequency. An oscilloscope was used to check the voltage and frequency. In order to make a Pd–ZnO sensor device, a 5 µL of Pd–ZnO NWs suspension was carefully placed between the electrode gap and was left to dry under an applied electric field. It took over 30 min to completely dry.

### 2.3 Simulations

Finite element method was employed in order to generate the electric field pattern of planar electrodes, using COMSOL Multiphysics. The design involved electrodes with significantly greater length (*z*-axis) than thickness (*y*-axis) and width (*x*-axis), resulting in a uniform electric field pattern along the electrode length. Therefore, we formulated a 2D geometry to produce the electric field distribution along the *x* and *y* axis, to optimize the computational power needed for simulations. The width and thickness of the electrodes were 1 cm (*x*-axis) and 100 nm (*y*-axis), respectively, while the gap between the electrodes was 50 µm (along *x*-axis). The applied voltage was 15 V was applied. For simulations, meticulous meshing parameters were established. Specifically, we set the minimum (2.5 nm) and maximum element sizes (2 µm), and an element growth rate and a resolution were chosen to be of 1.2 and 2, respectively, to ensure a balance between accuracy and computational efficiency.

To ensure operation within the frequency range where the DEP is positive, we analyzed the dielectrophoretic force equation to determine the corresponding frequency-dependent behavior of the dielectrophoretic force. Although the morphology of particles resembles more to cylinders, there is no simple closed-form analytical expression for calculating the dielectrophoretic force of a finite cylindrical wire.^19,20^ Therefore, we approximate the NWs as prolate ellipsoids. This approximation is reasonable for high-aspect-ratio NWs because it captures their elongated shape and the dominant polarization along the NW length. It also allows the dielectrophoretic force to be calculated analytically while accounting for the NW aspect ratio and dielectric properties, without requiring a full electromagnetic simulation of the actual cylindrical geometry. We recognize that a prolate ellipsoid is not identical to a cylindrical NW, particularly at the NW ends, and this difference can lead to some deviation in the calculated dielectrophoretic force due to differences in the local electric-field distribution. Nevertheless, for the high-aspect-ratio NWs considered in this work, the prolate-ellipsoid approximation provides a reasonable estimate of the dominant DEP behavior.

For a prolate ellipsoid, the dielectrophoretic force is given by the following equation^19^1
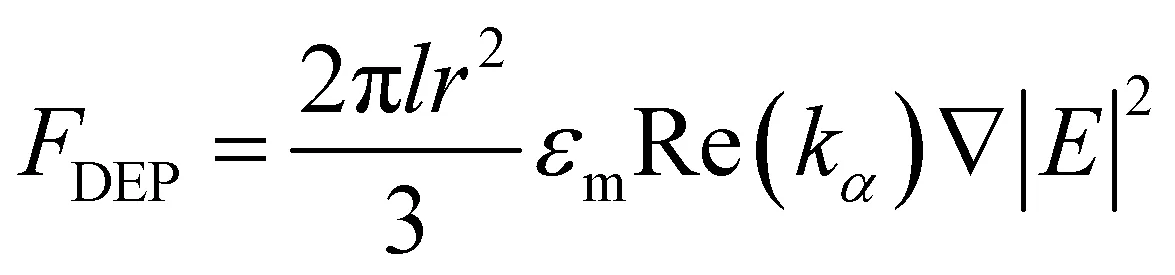
where *k*_*α*_ represents the Clausius–Mossotti Function (CMF) given by,2
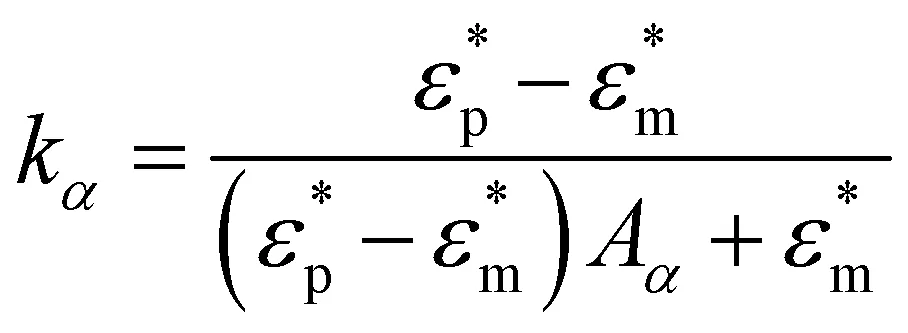
here *r* and *l* are the radius and length of the NW, respectively, *ε*_m_ is the medium's permittivity, *E* is the electric field strength and *A*_*α*_ represents the depolarization factor along the short and long axis of the NWs, which depends on the length and width of the NWs.^19^ The complex permittivity of the particle and medium is represented as 
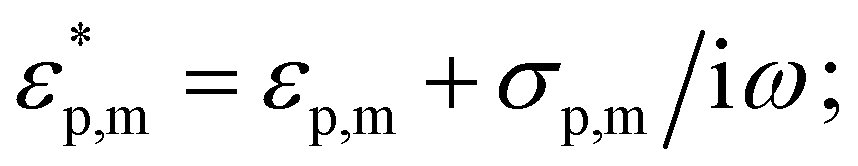
 where *σ*_p,m_ and *ε*_p,m_ are the conductivity and permittivity of the particle and suspended medium, respectively, and *ω* is the frequency of the applied field.

In order to calculate the CMF for ZnO and Pd, MATLAB was used to perform the required calculations. The electrical conductivity and permittivity of the constituent materials were specified based on the values reported in the literature. The permittivity of free space was taken as *ε*_0_ = 8.85 × 10^−12^ (F m^−1^). For ZnO, the electrical conductivity and relative permittivity were set to *σ*_p_ = 25 µS cm^−1^ and *ε*_p_ = 10*ε*_0_, respectively.^21^ For Pd NPs the corresponding values were *σ*_p_ = 9.5 × 10^10^ µS cm^−1^ and *ε*_p_ = 6.25*ε*_0_, respectively.^22^ For water, the electrical conductivity and permittivity were taken as *σ*_m_ = 1 µS cm^−1^ and *ε*_m_ = 78*ε*_0_, respectively.^22^

### 2.4 Humidity sensing experimental setup

The experimental setup of humidity sensing consists of five distinct salt boxes with varying humidity levels. The boxes that make up our humidity sensing system contain a supersaturated solution of various salts, such as LiCl, MgCl_2_, Mg(NO_3_)_2_, NaCl, and KNO_3_, which correspond to relative-humidity (RH) levels of 11%, 33%, 53%, 75%, and 97%. The electrical measurements of the device were taken using a Keithley multimeter and impedance analyzer.

The experimental setup used for the humidity-sensing measurements is schematically illustrated in Fig. 1. In the humidity sensing experiments, each RH level calibrated before measuring the sensor response. For this purpose, we prepared saturated salt solutions of various salts, such as LiCl, MgCl_2_, Mg (NO_3_)_2_, NaCl, and KNO_3_. Each saturated salt solution was placed in a suitable airtight chamber, which was then carefully sealed and allowed to equilibrate for a sufficient period to establish a stable humidity environment. The sensing device under test was subsequently placed inside boxes above the solution, ensuring that it did not come into direct contact with the liquid. At room temperature, each saturated salt solution establishes a known equilibrium RH within the sealed chamber. The RH inside the chamber was monitored using a calibrated reference hygrometer to ensure that the desired humidity level was accurately established and maintained during the measurements.

**Fig. 1:**
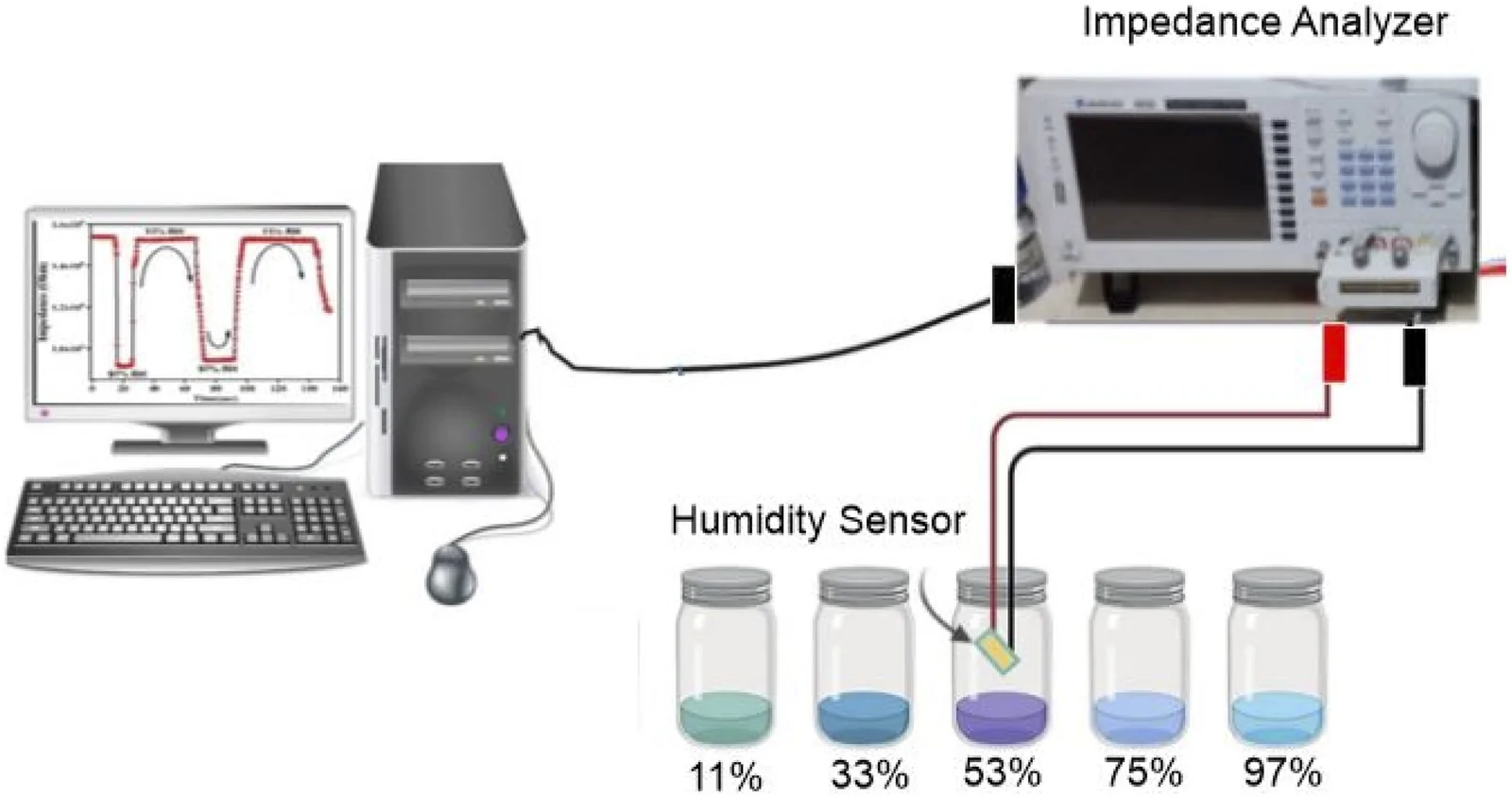
Schematic illustration of the experimental setup of humidity sensing.

## 3. Results and discussion

The SEM image in Fig. 2(a) depicts the fabricated ZnO NWs, exhibiting a distinct wire-like morphology. Analysis of the SEM image reveals that NWs have an average length and width measuring 9.51 µm, 610 nm, respectively. SEM images were also taken for Pd–ZnO NWs and are shown in Fig. 2(b). In the high-resolution SEM image, small Pd NPs can be seen on the surface of ZnO NWs.

**Fig. 2:**
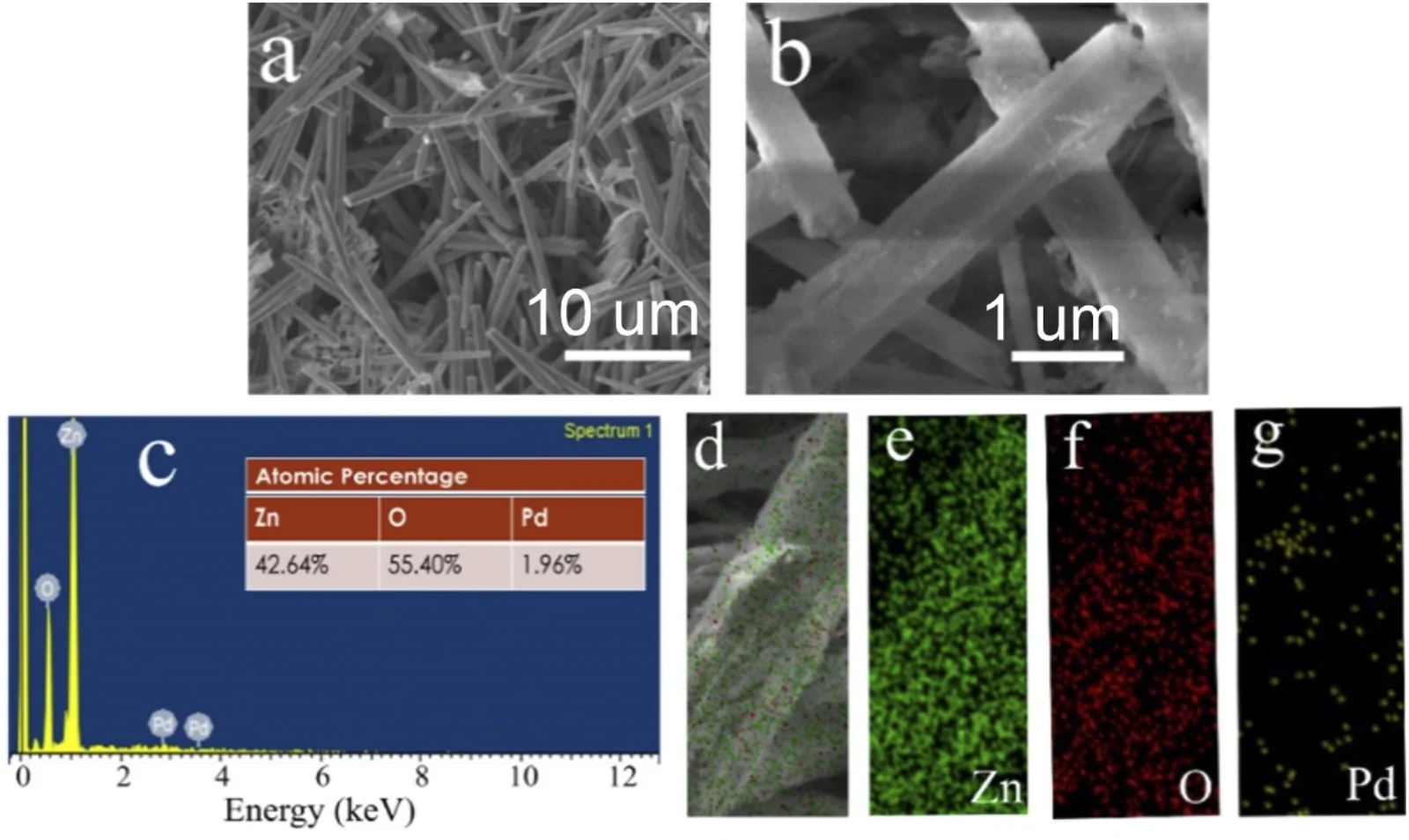
SEM image of ZnO NWs (a), and Pd–ZnO NWs (b). EDX spectrum (c) and EDX mapping images of Pd–ZnO NWs (d–g).

Fig. 2(c) depicts the EDX spectrum of Pd–ZnO NWs. The EDX confirms the successful coating of Pd on ZnO NWs, as evident by the presence of characteristic peaks corresponding to all the elements *i.e.* Zn, O, and Pd. Specifically, the characteristic peaks for Zn, O, and Pd are situated at 1.01 keV, 0.53 keV, and 2.8 keV, respectively. Additionally, the atomic percentage of Pd was observed to be 1.96% atomic percent. The EDS mapping analysis has been shown in Fig. 2(d–g). It validates the presence of Pd NPs on surface of ZnO NWs and further reveals the distribution of Pd across the ZnO NWs.

Fig. 3(a) depicts the XRD spectra of ZnO NWs and Pd-modified ZnO NWs. The 2*θ* values of ZnO peaks were obtained which match well with JCPDS 36-1451. The diffraction angles of peaks correlate to the (100), (002), (101), (102), (110), (103), (200), (112), (201), (004), and (202) indexes, respectively, which represent the hexagonal wurtzite phase of ZnO NWs.^18^ A weak feature, likely corresponding to (111) planes of Pd, appears between 40 and 50 (ref. 15 and 23) degree. The lower intensity of the Pd peak is attributed to the lower Pd concentration (1.96 atomic%), compared to ZnO, in the sample. This is similar to findings from a previous studies with similar concentrations of Pd and ZnO.^24,25^ Nonetheless, the high-pitched and sharp diffraction peaks show the good crystalline quality of ZnO NWs.^26^

**Fig. 3:**
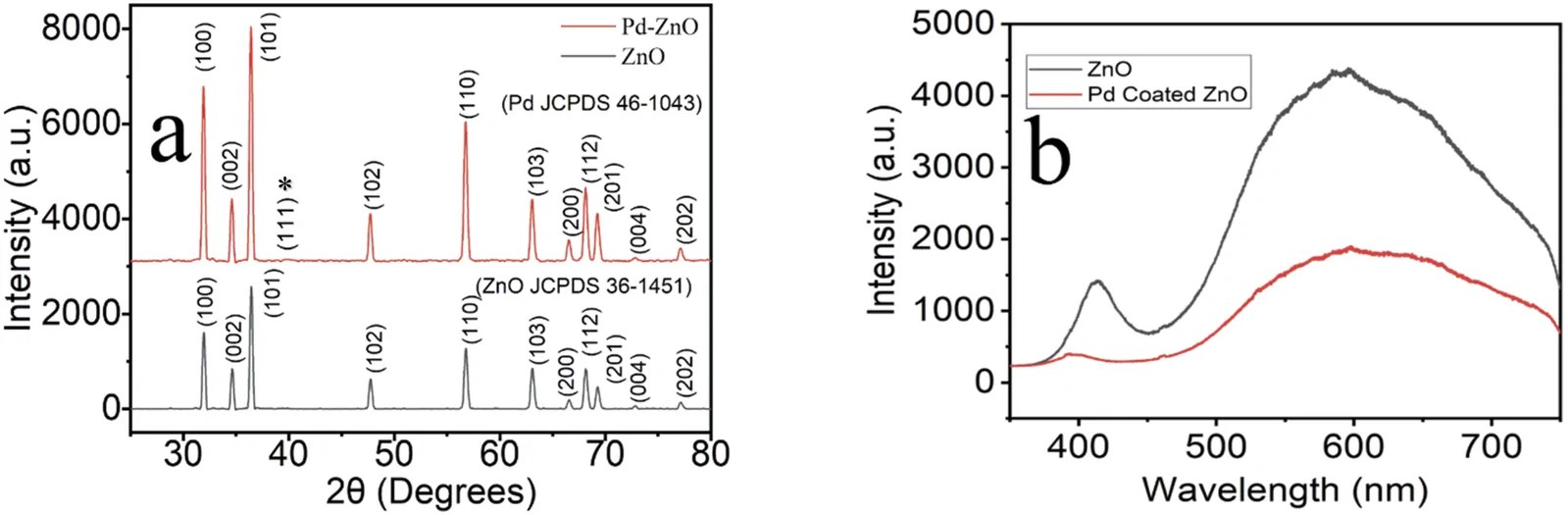
XRD pattern of ZnO and Pd–ZnO NWs (a). PL spectra of ZnO and Pd–ZnO NWs (b).

Fig. 3(b) shows the PL spectra of ZnO and Pd-modified ZnO NWs. The two separate emission bands of the ZnO material demonstrated a typical PL behavior. The UV region around 410 nm corresponded to free-excitonic recombination and near-band edge emission. ZnO's intrinsic defects, such as oxygen vacancies or other structural defects were located in the center of the visible region at a wavelength of about 590 nm.^27^ For Pd–ZnO, the UV emission of the Pd–ZnO NWs was not as intense as that of the ZnO NWs. This could be due to the fact that the excited electrons may have migrated into the Pd NPs, reducing the electron–hole recombination.^28^ The movement of electrons from the ZnO conduction band to Pd NPs also causes a decrease in intensity of defect emission. This outcome suggests that Pd might aid in lowering the oxygen vacancies in ZnO crystallites.^29,30^

### 3.1 Deposition of NWs

As evident from eqn (1), the direction of the dielectrophoretic force is determined by the sign of the real part of the CMF. A positive CMF results in positive dielectrophoretic force, driving the NW toward regions of higher electric-field intensity, whereas a negative CMF leads to negative dielectrophoresis, causing the NW to move toward regions of lower electric-field intensity. Thus, the CMF determines whether the NWs migrate toward or away from the electrode gap, where the electric-field intensity is highest. The CMF of ZnO NWs and Pd NPs as a function of frequency is shown in Fig. 4(a and b). The CMF for ZnO reaches a maximum value of 2.5 at frequencies up to 1 MHz. Above 1 MHz, the CMF gradually decreases, reaching zero at around 10 MHz and becoming negative at higher frequencies. In the case of Pd, the CMF remains positive up to 10^17^ Hz. Therefore, at the frequency used in this work (1 MHz), the CMF remains positive for both ZnO and Pd which will drive NWs in the higher field region between the electrodes.

**Fig. 4:**
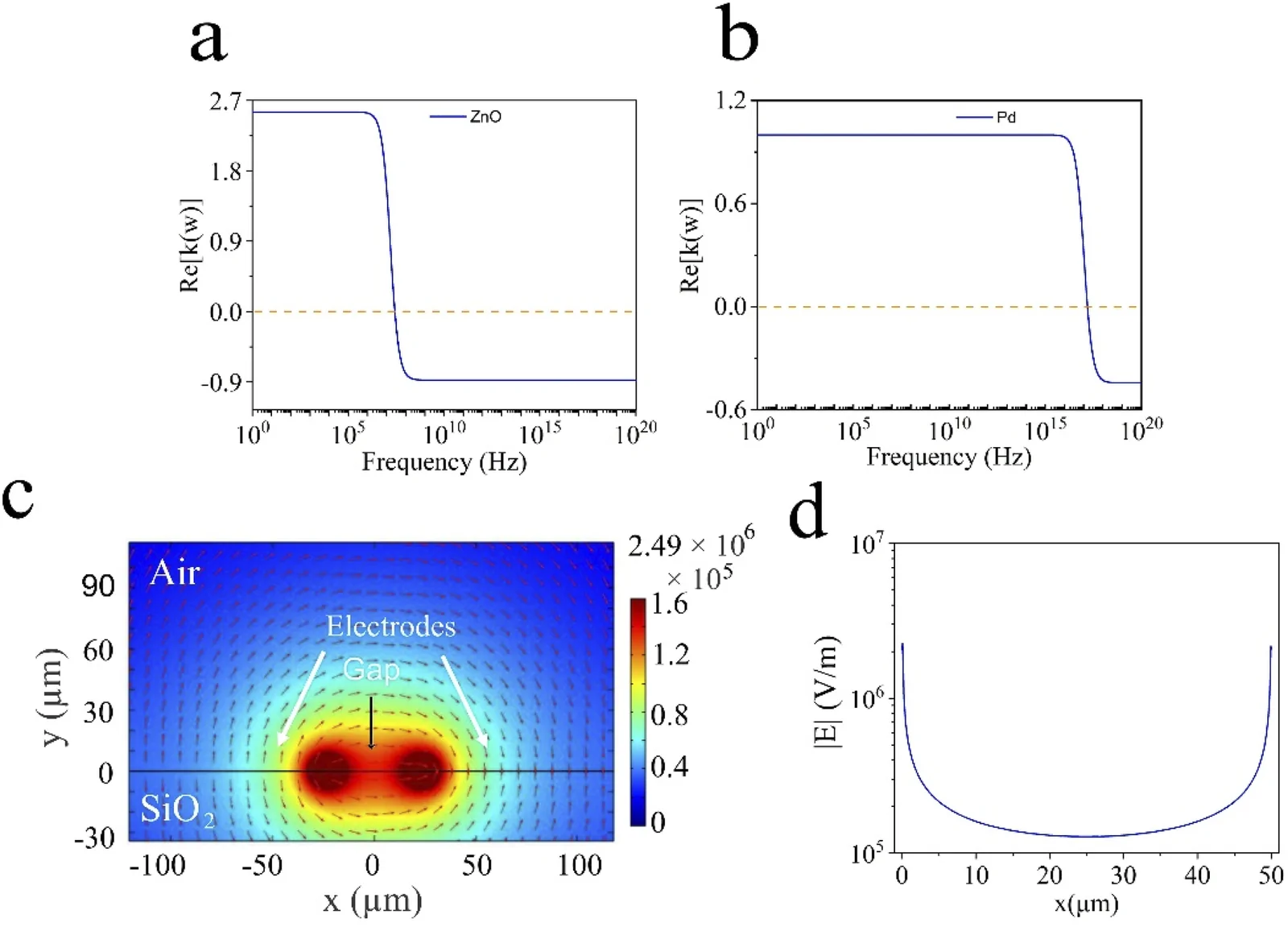
Calculated CMF as a function of frequency for ZnO (a) and Pd (b). 2-D field pattern of the device (c), and the line plot of the electric field at 100 nm above the substrate in the electrode gap (d).

Another factor that significantly affects the dielectrophoretic force is the electric field gradient, as indicated by eqn (1). In order to elucidate the electric field distribution between the planar electrodes, we carried out the finite element-based simulations. Fig. 4(c) shows the pattern of the electric field created by the simulations. The electric field strength is highest near the electrode edges due to field convergence, and it gradually decreases with distance from the electrodes along the *x*- and *y*-axes, within the electrode gap, or above the electrodes. Fig. 4(b) depicts the line plot of the electric field inside the electrode gap, 100 nm above the substrate's surface. The field strength varies from 0.6 × 10^4^ V m^−1^ in the middle of the electrode gap to 1.2 × 10^6^ V m^−1^ near the electrode edge. These values are considerably higher than the critical field needed to overcome Brownian motion and Buoyancy force.^18^ This implies that NWs close to the electrode gap are trapped by DEP. Consequently, the electric field dictates the deposition of NWs.

The SEM image shown in Fig. 5(a) confirms that a large number of Pd–ZnO NWs were trapped within the electrode gap. Specifically, from the image, it is confirmed that a higher concentration of NWs is present within the electrode gap as compared to the surface of electrodes. This variation in the concentration of NWs highlights the importance of dielectrophoretic force in effective deposition of NWs between electrodes.

**Fig. 5:**
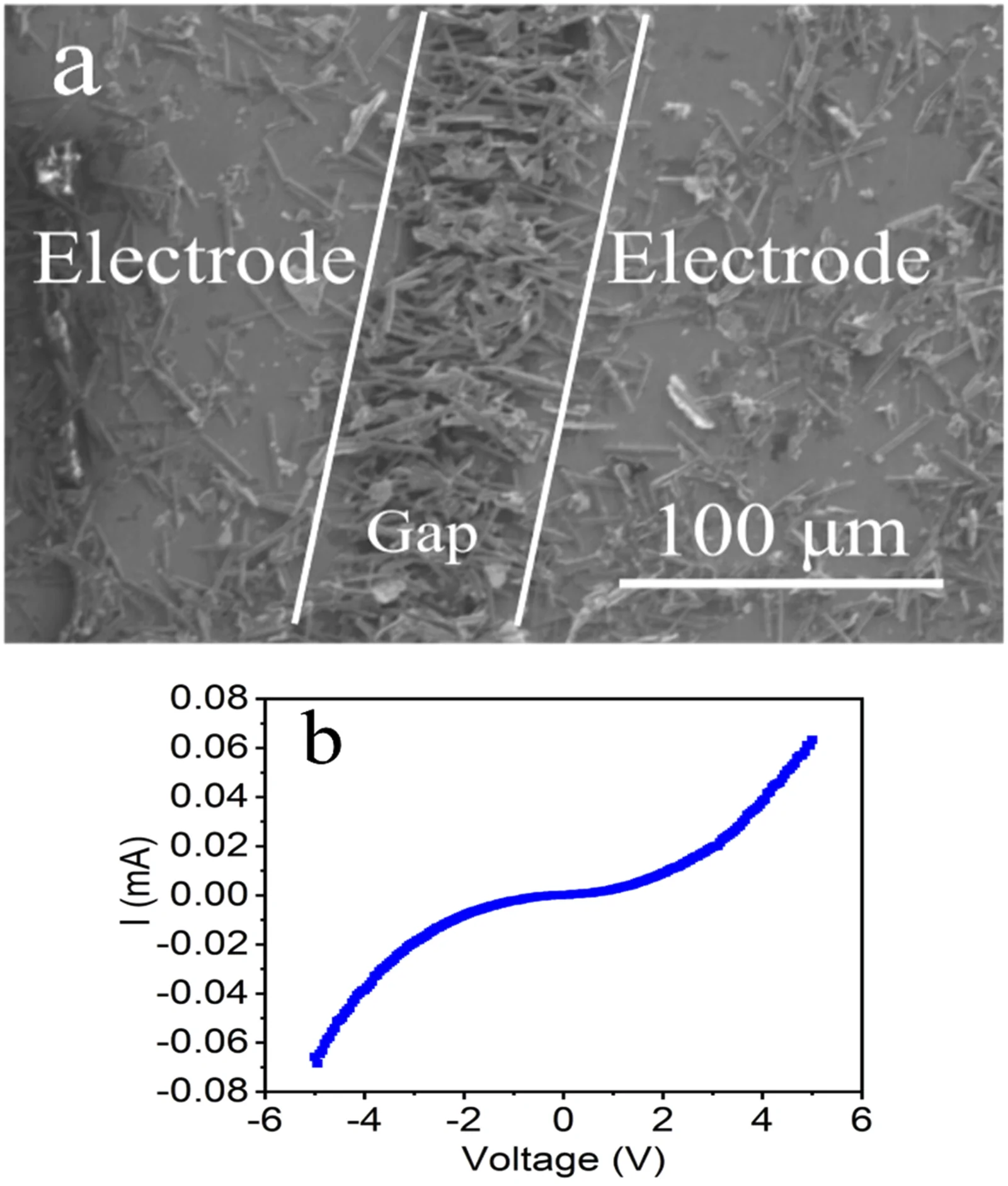
SEM image of device after deposition (a) and the *I*–*V* curve of the sensor (b).

The electrical transport properties of the Pd-decorated ZnO NWs were evaluated by measuring the current–voltage (*I*–*V*) characteristics under an applied bias ranging from −5 V to +5 V (Fig. 5(b)). The non-linear *I*–*V* profile indicates non-ohmic charge transport, with current increasing progressively alongside the magnitude of the applied bias. The relatively low current near zero bias stems from restricted carrier transport across the semiconductor NW network, whereas the enhanced current at higher bias reflects field-assisted charge transport.^31,32^ This observed non-linearity arises from the combined effects of the semiconducting nature of ZnO, surface and defect states, NW–NW junction barriers, and interfacial Schottky barriers at the Pd/ZnO interfaces.

### 3.2 Humidity sensing

In order to check the humidity sensing capabilities of the device, impedance measurements were taken at the relative humidity range of 11% to 97%. Fig. 6(a) shows the frequency dependance of the real part of the impedance. There is a clear decrease in impedance with increase in the humidity levels. Notably, the sensor's impedance is relatively strongly influenced by % RH at lower frequencies than at higher ones. This can be attributed to the rapid changes in the electric field direction at higher frequencies, which leads to an inability for water polarization to keep up, resulting in a smaller dielectric constant and relative humidity independence. At lower frequencies, the slower reversal of the electric field direction allows greater space-charge polarization of the adsorbed water molecules, resulting in a pronounced impedance shift. Consequently, the system exhibits frequency-dependent capacitance at lower frequencies, whereas at higher frequencies the rapid field oscillations prevent effective polarization, leading to negligible and frequency-independent capacitance.^18,33^

**Fig. 6:**
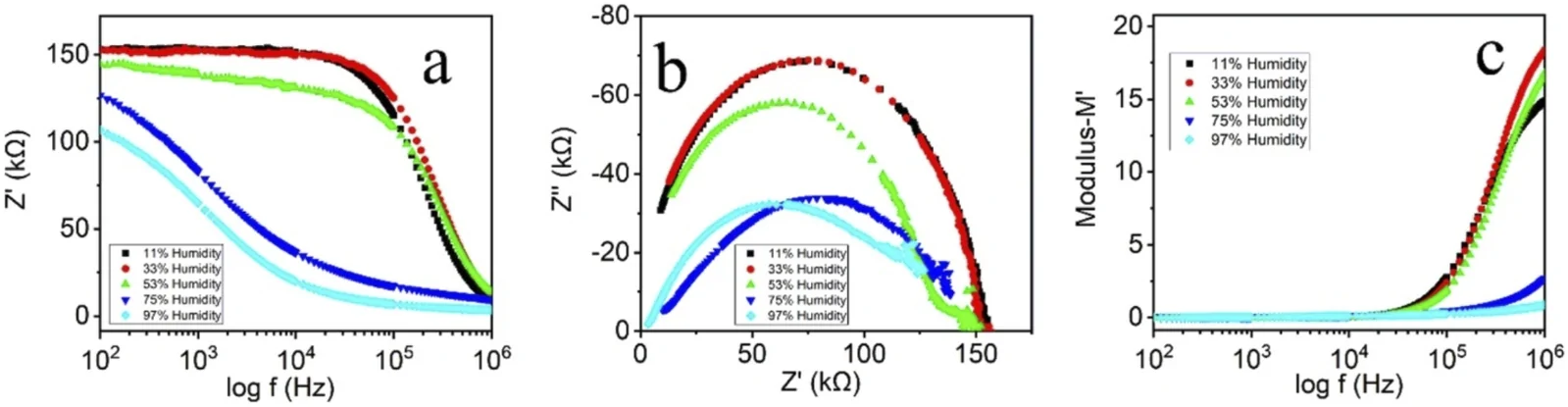
Frequency *vs.* real impedance plot (a); Nyquist plot of the real *versus* imaginary impedance (b); frequency *vs.* real modulus plot (c).

The correlation between the real and imaginary parts of the impedance at different relative humidity levels is depicted in Fig. 6(b). It can be seen in the graph that clear semicircles have appeared. The bulk resistance of the device was obtained from the intercept of the semicircle with the real axis. As shown in the figure, the bulk resistance decreases with increasing % RH, dropping from 155 kΩ at 11% RH to 124 kΩ at 97% RH.

Fig. 6(c) present the electric modulus analysis of Pd–ZnO NW-based device at different relative humidities. Modulus can also be referred to as electric modulus M*(*ω*), is a useful concept for understanding relaxation processes in Pd–ZnO NWs. It is mathematically defined in terms of the complex impedance as:^32^

3 M*(*ω*) = *M*′ + i*M*″(*ω*) = i*ωC*_0_Z*(*ω*)

where *M*′(*ω*) and *M*″(*ω*) are the real and imaginary parts of the modules, respectively, *ω* is the angular frequency and *C*_0_ is geometrical capacitance parameter *b*.^34^

The electric modulus emphasizes localized polarization and suppresses the influence of electrode polarization and dc conduction. In Fig. 6(c)*M*′(*ω*) is close to zero at low frequencies for all humidities, which is a signature of large low-frequency permittivity arising from long-range charge transport and electrode effects.^35^ At higher frequencies *M*′(*ω*) rise as localized relaxations become active. For low to moderate RH (11 → 33 → 53%), the modulus response becomes more pronounced, indicating an increase in localized polarizable hopping species (adsorbed water molecules and associated proton/hydrated ion hopping). However, at the highest humidities (75–97%) the modulus is strongly suppressed across the frequency range; this behavior is consistent with the dominance of long-range ionic conduction (formation of continuous water layers) that increases the permittivity and thereby reduces the modulus.

The presence of Pd plays a critical role in these trends. Pd NPs on the ZnO NWs surface provide catalytic sites for water dissociation, producing mobile protons that contribute to localized hopping conduction.^15,16^ In addition, Pd modifies the surface electronic structure of ZnO, as illustrated in Fig. S1 of the SI, thereby enhancing charge transfer and facilitating faster polarization–depolarization processes.^31^ As a result, Pd–ZnO NWs exhibit stronger relaxation features and a more pronounced suppression of the modulus at high humidity compared to undoped ZnO. Thus, the introduction of Pd significantly amplifies the humidity-driven dielectric relaxation processes observed in the modulus spectra.

Fig. 7 illustrates the impedance response of the Pd–ZnO NW-based humidity sensor. As shown in Fig. 7(a), the impedance decreases monotonically with increasing RH. At low humidity, only chemisorbed water molecules are present on the ZnO surface, resulting in high impedance due to limited charge carriers. With increasing RH, additional physiosorbed water layers form, enabling proton hopping *via* the Grotthuss mechanism,^36^ which drastically enhances ionic conduction and reduces impedance. The incorporation of Pd significantly accelerates this process by providing catalytic sites for water dissociation, thereby generating more protons and further lowering impedance compared to undoped ZnO.^37^

**Fig. 7:**
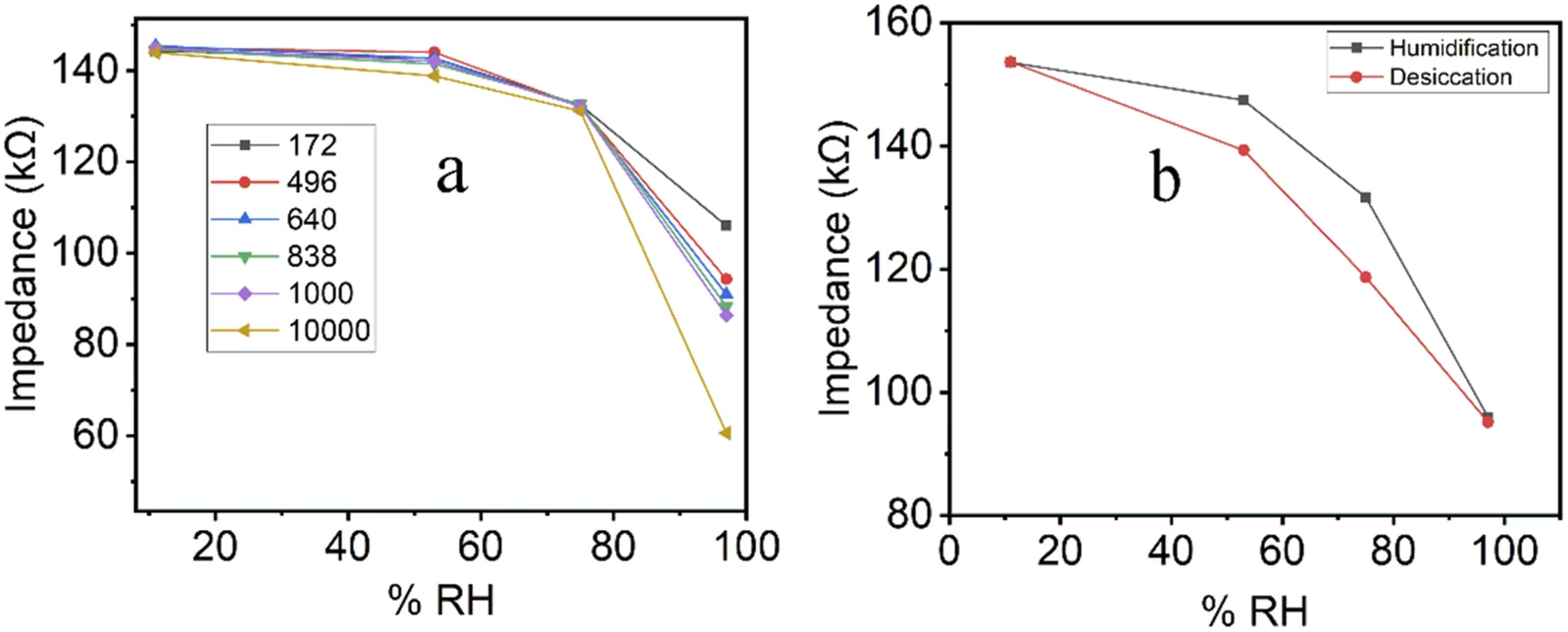
Impedance *vs.* relative humidity plot (a) and hysteresis of the sensor (b).

The hysteresis plot of the device is shown in Fig. 7(b). Hysteresis is an important parameter, and it occurs because of the differences in the adsorption and desorption of water molecules onto the surface of the sensor. As evident from Fig. 7(b), the black line spanning from 11% to 97% relative humidity (RH) shows the adsorption of water molecules, while the red line depicts the desorption process from 97% back to 11% relative humidity. The impedance during the adsorption phase surpasses that of desorption. This discrepancy arises from the inherently faster adsorption kinetics compared to desorption. Adsorption, being exothermic and spontaneous, occurs swiftly without necessitating external energy inputs.^38^ Conversely, desorption is slower in the Pd–ZnO system due to the substantial bonding energies between water molecules and surfaces, making the removal process more difficult. The magnitude of the shift in the adsorption–desorption curve, known as humidity hysteresis, serves as a testament to the device's stability.^39^ Hysteresis can be measured using the following equation:4
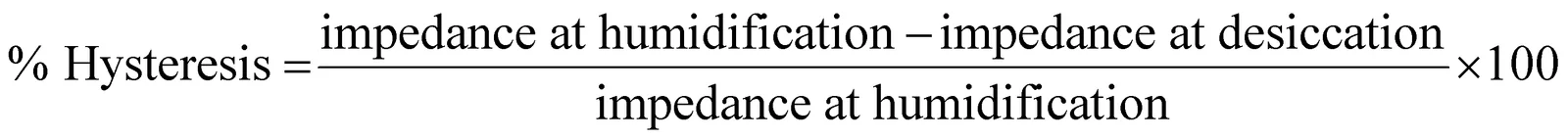


The sensor exhibits minimal hysteresis of 5.5%, confirming its reversibility and stability under cyclic humidity variations. The improved reversibility in Pd–ZnO NWs is attributed to Pd-induced modifications of the surface electronic structure, which reduce trapping of water molecules and facilitate faster desorption. These results highlight the superior sensitivity and reliability of Pd–ZnO NW humidity sensors compared to pure ZnO counterparts.

Fig. 8 shows the response and recovery time curves of the sensor between two humidity levels, 11% RH and 97% RH. The response time was determined when the device transitioned from a lower to a higher RH level and is defined as the time required to reach 90% of the total resistance change. Conversely, the recovery time was measured when the sensor returned from higher to lower RH level using the same criterion. These parameters reflect the dynamics of water molecule adsorption and desorption on the sensing surface: response time corresponds to adsorption, while recovery time corresponds to desorption.^18^ As discussed earlier, desorption is slower than adsorption in the present Pd–ZnO NWs system, which may be attributed to the stronger interaction of water molecules with the Pd–ZnO NWs surface and the time required for the adsorbed water layers to desorb during the recovery process.^38^ From the plot, the sensor exhibits an average response time of 5 s and an average recovery time of 8 s.

**Fig. 8:**
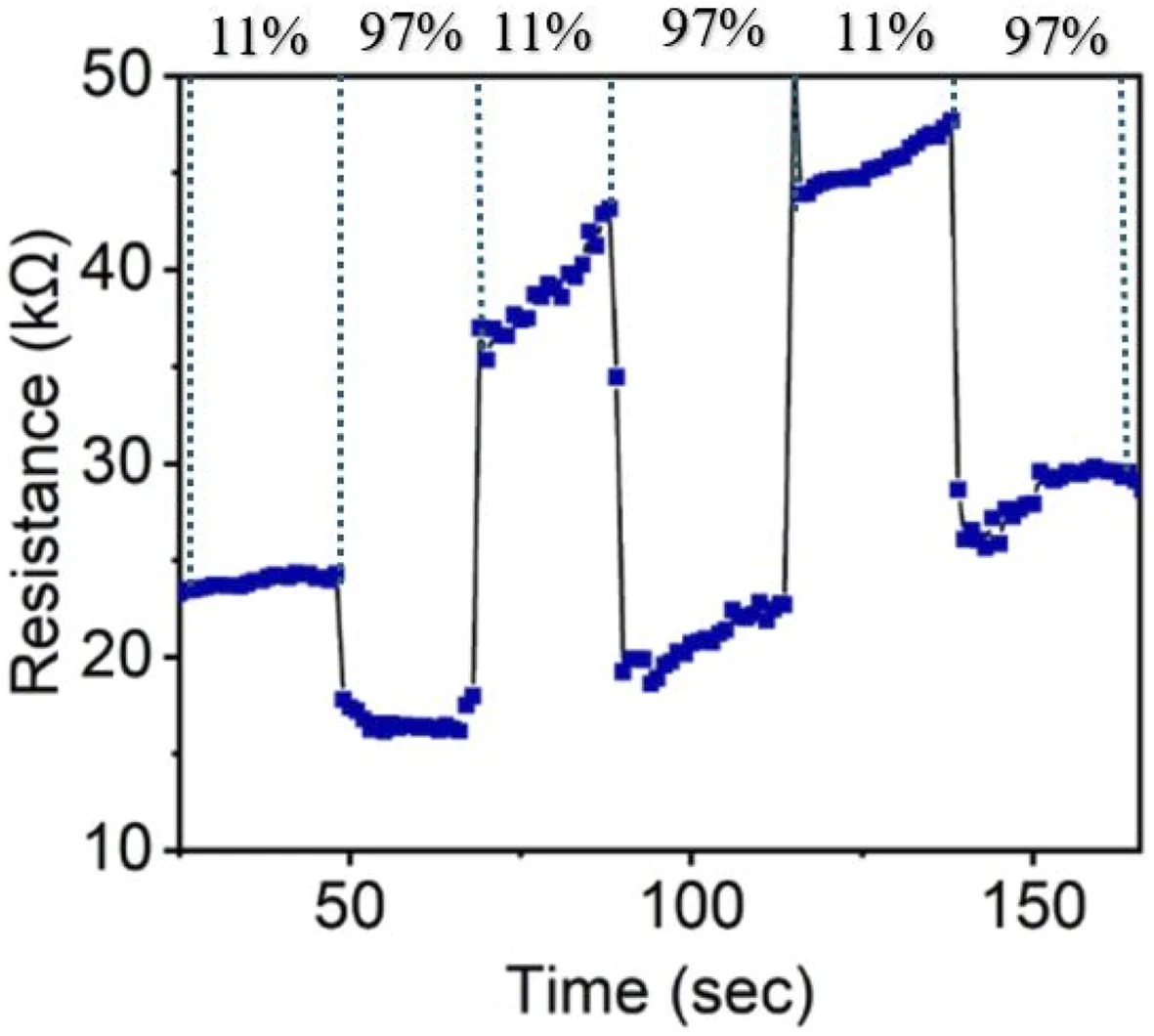
Curve shows the response and recovery times of the Pd–ZnO sensor.

Another key parameter for evaluating the performance of a humidity sensor is its sensitivity, which underscores the device's ability to detect even the subtlest changes in humidity levels. It is defined as:5
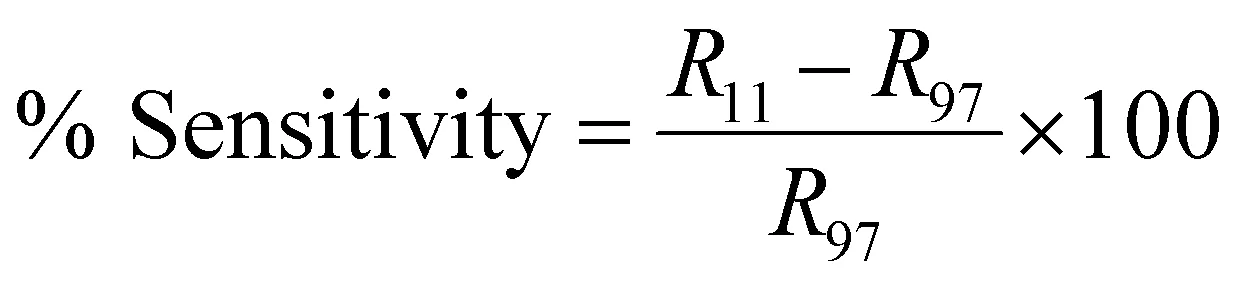


The resistances of the device at lower and higher humidity values are denoted as *R*_11%_ and *R*_97%_, respectively.^40^ Our sensor exhibits a sensitivity of approximately 54%, indicating its high responsiveness to humidity fluctuations.

Table 1 provides a detailed comparison of our sensor with previously reported ZnO-based devices. In contrast to earlier studies, our sensor exhibits remarkably faster response and recovery times, clearly highlighting its superior performance. This advancement can be attributed to several key factors. First, the deposition of ZnO NWs was improved through DEP, a process that ensured a highly uniform alignment and distribution of NWs, thereby creating a larger number of active sites for water molecule adsorption and desorption. Second, the incorporation of Pd introduced both electronic and catalytic enhancements: electronically, Pd facilitated efficient charge transfer at the ZnO–Pd interface,^31^ while catalytically, it promoted surface reactions with water molecules,^37^ assisting the water dissociation which in turn leads to accelerated sensor kinetics. Together, these design strategies synergistically improved the efficiency, sensitivity, and overall responsiveness of the device, positioning it as a highly competitive alternative to conventional ZnO NW-based humidity sensors.

**Table 1:** Comparison of the findings of this study with those reported in the literature

Material	Humidity	Response time (s)	Recovery time (s)	Deposition method	Reference
ZnO NWs	12–96%	1980	31	Vertically grown NWs	41
Sn doped ZnO NRs	40–90%	230	30	Sol–gel immersion method assisted by sonication	42
ZnO NWs	1.73–82.13%	202	57.4	Randomly growth of NWs between electrodes	43
ZnO–TiO_2_ core–shell nanocomposites	11–95%	774.9	19.7	*In situ* growth of ZnO/TiO_2_ nano composites on glass surfaces	44
Er–ZnO NPs	11–95%	32	39	Spin coating	45
ZnO–SiO_2_ NPs	11–95%	50	100	Spin coating	46
ZnO–PVA NWs	40–90%	40	60	Hydrothermally grown NWs	47
Cellulose/ZnO nanocomposite	5–98%	26	53	Drop casting	48
ZnO/GQDs nanocomposite	11–43%	30	160	Spin coating	49
ZnO NWs	11–97%	17	9	DEP	18
Pd–ZnO NWs	11–97%	5	8	DEP	Present work

## 4. Conclusion

In conclusion, the present study demonstrates the successful use of DEP assembly of Pd-modified ZnO NWs for the fabrication of a high-performance humidity sensor. The simulation and experimental results confirm that DEP provides an effective means of assembling and positioning the NWs within the electrode gap, with an applied voltage of 15 V providing sufficient electric force for efficient assembly. The incorporation of Pd significantly enhanced the humidity-sensing performance of the ZnO NW-based device. This improvement can be attributed to the catalytic role of Pd which facilitates water dissociation, together with the formation of a Schottky junction at the Pd/ZnO interface and the associated band bending. The resulting sensor exhibited a sensitivity of 54%, a response and recovery times of 5 and 8 s, respectively, and notably low hysteresis of 5.5%. The overall sensing performance compares favorably with previously reported ZnO-based humidity sensors, highlighting the beneficial effect of Pd modification and the DEP-assisted assembly approach. These findings demonstrate that the combination of Pd-functionalized ZnO NWs and dielectrophoretic assembly offers a promising strategy for the development of fast, sensitive, and reliable humidity sensors.

## Conflicts of interest

There are no conflicts to declare.

## Supplementary Material

RA-OLF-D6RA05014J-s001

## Data Availability

Data that supports the findings of this study will be available, upon request. Supplementary information: schematic illustrations of the humidity-sensing mechanism and the energy-band diagram of the ZnO–Pd heterostructure. See DOI: https://doi.org/10.1039/d6ra05014j.
