# Supplementary material for: Dielectrophoretic deposition of palladium-modified zinc oxide nanowires for high-performance humidity sensors
Source: RSC Adv. 2026 Oct 1;16(46):59700–9. doi: 10.1039/d6ra05014j (PMC13629750; doi:10.1039/d6ra05014j)
Supplement: Supplementary file 1 [file RA-016-D6RA05014J-s001.pdf]

Supplementary Information (SI) for RSC Advances.
This journal is © The Royal Society of Chemistry

# Dielectrophoretic Deposition of Palladium- Modified Zinc Oxide Nanowires for High-Performance Humidity Sensors

**Kainat Farooq,[1] Muhammad Tayyab Zahoor,[1] Zahid Imran,[2] Waqqar Ahmed,[1*]**

[1] *Materials Laboratory, Department of Physics, COMSATS University Islamabad, Park Road, 45500, Pakistan*

[2] *Catalysis and Sensing Materials Laboratory, Department of Physics, COMSATS University, Islamabad, Park Road, 45500, Pakistan*

*corresponding author's email: w.ahmed@comsats.edu.pk; Tel: +925190495305

Fig. S1 depicts the humidity sensing mechanism of Pd-ZnO NWs. At lower humidity levels, water molecules chemisorbed on the nanowire surface, and during this stage discontinuous proton conduction leads to a higher impedance value. As humidity increases, physisorption occurs, increasing proton conduction by facilitating proton hopping through the formation of multiple physiosorbed layers. As a result, impedance decreases continuously with increasing humidity [1, 2]. The presence of Pd on ZnO is expected to enhance the humidity sensing capabilities of the sensor. This is due to the fact that in the Pd–ZnO heterostructure, a Schottky junction forms at the interface, creating a depletion region (Fig. S1(b)). At higher humidity levels, enhanced proton conduction across this region reduces resistance.

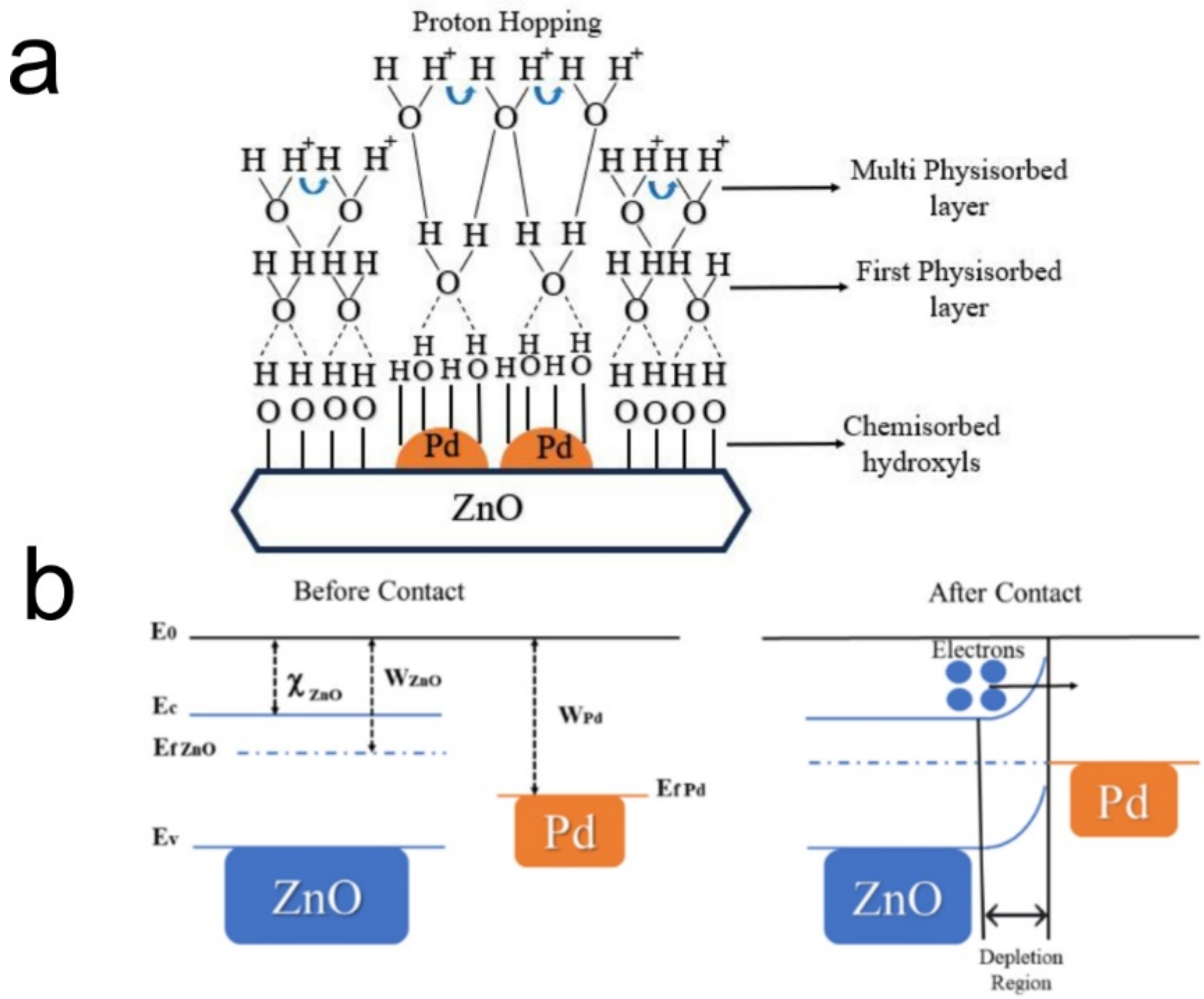


**Figure S1.** Schematic of the water adsorption on Pd-ZnO humidity sensor (a), and energy band diagram of ZnO-Pd heterostructure (b).

## References

[1] L. Kumari, U. Kumar, L. Sinha, O. Prasad, B. Yadav, M. Gupta, Surface modification and characterization of h-BN-doped PVP thin film and its application as humidity sensor with theoretical DFT calculations, Chemical Papers, 75 (2021) 4055-4068.

[2] Y. Li, M. Jiao, H. Zhao, M. Yang, Humidity sensing properties of the composite of electrospun crosslinked polyelectrolyte nanofibers decorated with Ag nanoparticles, Sensors and Actuators B: Chemical, 273 (2018) 133-142.
